# Bevacizumab in Platinum-Sensitive Recurrent Epithelial Ovarian Cancer: A Risk-Stratified Analysis

**DOI:** 10.3390/ph18060850

**Published:** 2025-06-06

**Authors:** İrem Öner, Pınar Karaçin

**Affiliations:** 1 Department of Medical Oncology, Dr. Abdurrahman Yurtaslan Ankara Oncology Research and Training Hospital, Ankara 06200, Turkey; 2 Department of Obstetrics and Gynecology, Dr. Abdurrahman Yurtaslan Ankara Oncology Research and Training Hospital, Ankara 06200, Turkey; pinarkaracin@gmail.com

**Keywords:** platinum sensitive, recurrent epithelial ovarian cancer, bevacizumab, high risk, low risk, cytoreductive surgery, location of recurrence

## Abstract

**Background and Objectives:** Epithelial ovarian cancer (EOC) is a complex disease characterized by heterogeneous clinical, pathological, and molecular features. Diagnosed frequently at advanced stages, it presents significant challenges in treatment due to the risks of resistance and recurrence. While bevacizumab, by inhibiting angiogenesis, offers a valuable therapeutic option for platinum-sensitive recurrent ovarian cancer (PSROC), its impact on overall survival (OS) remains incompletely understood. This retrospective study aims to compare treatment responses in high- and low-risk groups of patients with PSROC and to evaluate the effects of bevacizumab on survival within these risk strata. **Materials and Methods:** This retrospective study included patients diagnosed with International Federation of Gynecology and Obstetrics (FIGO) stage III and IV EOC who received chemotherapy or chemotherapy plus bevacizumab for platinum-sensitive recurrence. Patients were classified according to risk groups, and their clinicopathological characteristics were compared. Survival analyses and adverse events regarding risk groups and bevacizumab use were examined. In this study, the effect of bevacizumab on survival in patients with PSROC was evaluated for the first time according to risk stratification, and the relationship between treatment response and survival was investigated according to recurrence localization. **Results:** Of the 174 patients included in this study, 102 (58.6%) were classified as low risk and 72 (41.4%) were classified as high risk. Significant differences in survival were observed between the risk groups. In the low-risk group, progression-free survival and overall survival were markedly longer compared to the high-risk group. Median PFS was 13.7 months in the low-risk group and 10.8 months in the high-risk group (*p* = 0.007). Median OS was 36.5 and 23.5 months, respectively (*p* = 0.003). In low-risk patients, the addition of bevacizumab to chemotherapy significantly increased median PFS (13.5 months vs. 9.7 months, *p* = 0.029); however, this advantage did not translate into a significant overall survival benefit (39.4 months vs. 33.3 months, *p* = 0.669). Conversely, in the high-risk group, bevacizumab use provided significant benefits in both PFS and OS. Median PFS was 13.9 months in the bevacizumab group and 8.8 months in the control group (*p* < 0.001). Median OS was calculated as 36.5 and 23.2 months, respectively (*p* < 0.001). **Conclusions:** Our study is among the first to comprehensively compare the effectiveness of bevacizumab treatment in patients with platinum-sensitive recurrent ovarian cancer based on clinical risk groups and recurrence patterns using real-world data. The current literature lacks a comprehensive analysis that simultaneously evaluates these two critical parameters. In this respect, our study aims to contribute to developing more personalized treatment strategies for specific patient subgroups.

## 1. Introduction

Ovarian cancer ranks among the most prevalent gynecological malignancies worldwide, presenting as a heterogeneous disease characterized by diverse clinicopathological and molecular features [1,2]. Epithelial ovarian carcinoma (EOC) constitutes the most frequently encountered subtype of ovarian malignancies, accounting for approximately 90% of all cases [3]. The standard treatment protocol for patients diagnosed with EOC involves surgical cytoreduction followed by a combination of paclitaxel and platinum-based chemotherapy. However, the fact that approximately 70% of cases are diagnosed at advanced stages limits treatment options and negatively impacts prognosis by increasing the risk of treatment resistance and tumor recurrence over time [4]. Despite significant advancements in treatment strategies in recent years, EOC remains a substantial challenge in clinical oncology due to high recurrence rates [5,6,7]. Considering the critical role of angiogenesis in EOC progression, vascular endothelial growth factor inhibitors (VEGFs) present a significant treatment option [6,8].

Bevacizumab, a VEGF inhibitor, received approval for EOC treatment from the European Medicines Agency in December 2011 and the United States Food and Drug Administration in June 2018 [9]. Bevacizumab remains a crucial treatment option for patients unsuitable for poly(ADP-ribose) polymerase (PARP) inhibitors or those who would derive maximum benefit from platinum-based therapies, both in treatment-naive and recurrent settings. Current evidence suggests that adding bevacizumab to platinum-based chemotherapy in platinum-sensitive recurrent ovarian cancer (PSROC) may improve progression-free survival (PFS). However, it does not significantly impact overall survival (OS) [2,10]. However, subgroup analyses of the ICON7 and GOG-0218 trials, which evaluated the benefit of adding bevacizumab to first-line chemotherapy in treatment-naive patients, demonstrated a positive impact on overall survival (OS), particularly in high-risk patients [11,12]. Although bevacizumab has proven effective in treating PSROC, its influence on survival, considering varying clinical risk categories and recurrence sites, is not well established. This study seeks to determine bevacizumab’s effect on survival outcomes relative to risk groups and to delineate differences based on recurrence patterns. To our knowledge, no prior research has integrated these parameters in a holistic analysis using real-world data.

Evaluating therapeutic efficacy by stratifying patients into high- and low-risk groups is indeed a crucial approach for optimizing treatment response and minimizing adverse effects, especially in complex cases like advanced EOC. Risk-based decision making aims to improve clinical practice by contributing to the development of personalized treatment strategies, particularly in complex conditions such as advanced-stage EOC [13,14].

For these reasons, this study aimed to evaluate the effects of bevacizumab use in the treatment of patients diagnosed with PSROC. Based on factors such as clinical stage and residual disease status, we divided the patients into high- and low-risk groups and compared their treatment responses. Through this comparison, we examined the impact of bevacizumab on treatment efficacy and patient prognosis.

A distinctive aspect of our study is that it is among the first to comprehensively compare the effectiveness of bevacizumab treatment in patients with platinum-sensitive recurrent ovarian cancer based on clinical risk groups and recurrence patterns using real-world data. The literature lacks a thorough analysis that concurrently evaluates these two critical parameters. In this respect, our study aims to contribute to the development of more personalized treatment strategies tailored to specific patient subgroups.

## 2. Results

A total of 174 patients with platinum-sensitive recurrent ovarian cancer (PSROC) were analyzed, with 58.6% classified as low risk and 41.4% as high risk. The median age of the patients was 58 (range 28–85) years. Key distinctions emerged: high-risk patients more frequently presented with ECOG scores of 2 (29.2% vs. 4.9%, *p* < 0.001) and FIGO stage IV disease (*p* < 0.001) and underwent suboptimal cytoreduction (*p* < 0.001). The proportions of patients receiving chemotherapy and bevacizumab treatment were balanced across the risk groups (*p* = 0.451). The clinicopathological characteristics of the patients are summarized in Table 1. *p*-values were calculated to compare variables between groups, and a value of less than 0.05 was considered statistically significant.

**Figure 1:**
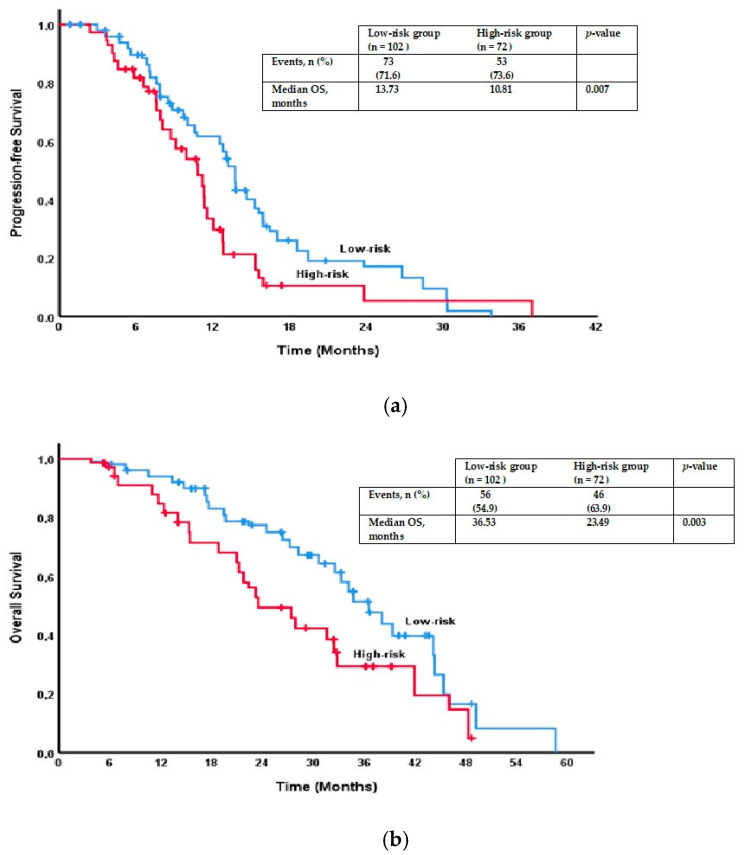
(**a**) Kaplan–Meier curves for progression-free survival between high- and low-risk groups. (**b**) Kaplan–Meier curves for overall survival between high- and low-risk groups.

Significant survival differences emerged after a median follow-up of 36.7 (95%CI: 33.46–39.87) months. Low-risk patients demonstrated a median PFS of 13.7 months versus 10.8 months in the high-risk group (*p* = 0.007; Figure 1a). Similarly, median OS was markedly longer in the low-risk group at 36.5 months compared to 23.5 months in the high-risk group (*p* = 0.003; Figure 1b).

Patients treated with chemotherapy plus bevacizumab had significantly improved PFS compared to chemotherapy alone (13.7 vs. 9.7 months, *p* < 0.001), while OS showed a non-significant trend in favor of bevacizumab (36.5 vs. 32.4 months, *p* = 0.178). Risk stratification further highlighted the survival benefit of bevacizumab, especially in the high-risk group. PFS and OS also varied based on surgical status and recurrence site. Median PFS was 11.3 months for suboptimal cytoreduction, 13.2 months for optimal cytoreduction, and 7.0 months for inoperable cases (*p* = 0.056). Corresponding median OS was 31.6, 36.5, and 18.8 months, respectively (*p* = 0.013). For recurrence site, median PFS was 13.0 months for locoregional, 10.6 months for distant, and 10.8 months for isolated lymph node recurrence (*p* = 0.030), with a corresponding median OS of 34.7, 31.6, and 21.2 months (*p* = 0.011). An interval of more than 12 months from the last platinum dose was associated with improved overall survival (OS) (36.5 vs. 27.9 months, *p* = 0.014) but not PFS (Table 2).

**Figure 2:**
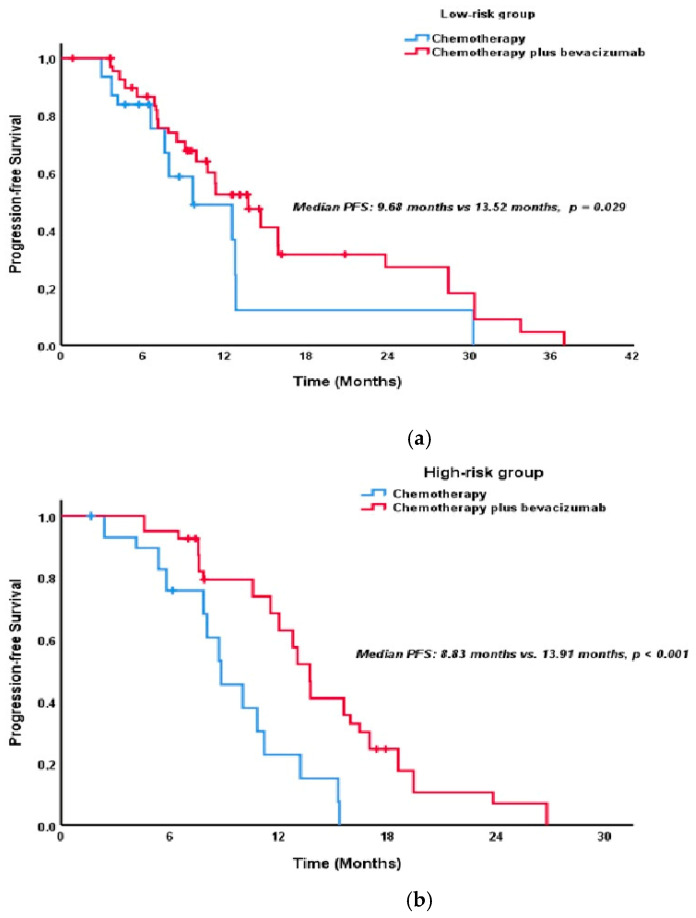
(**a**) Kaplan–Meier curves of PFS in the low-risk group, comparing chemotherapy alone versus chemotherapy plus bevacizumab. (**b**) Kaplan–Meier curves of PFS in the high-risk group, comparing chemotherapy alone versus chemotherapy plus bevacizumab.

**Figure 3:**
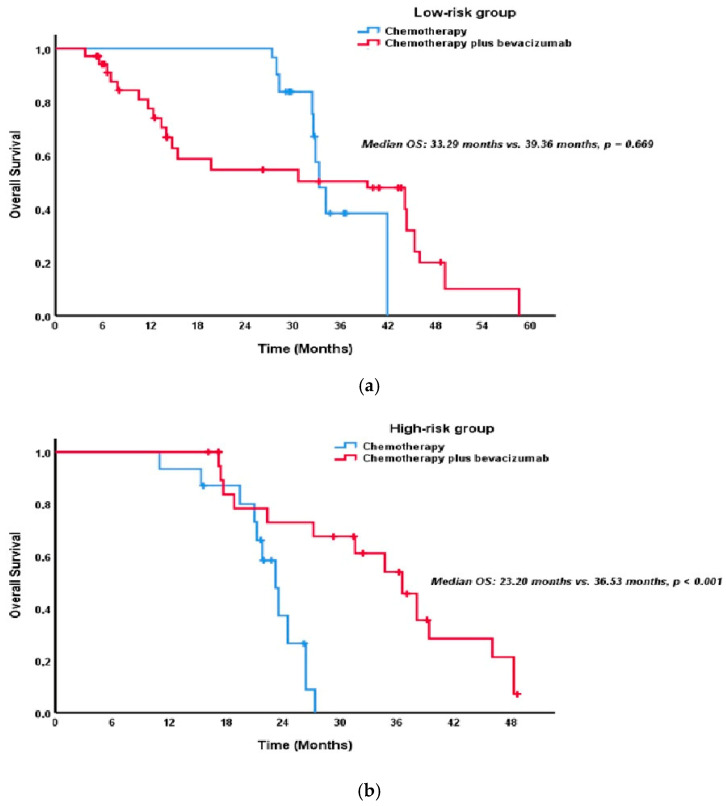
(**a**) Kaplan–Meier curves of OS in the low-risk group, comparing chemotherapy alone versus chemotherapy plus bevacizumab. (**b**) Kaplan–Meier curves of OS in the high-risk group, comparing chemotherapy alone versus chemotherapy plus bevacizumab.

Stratified by risk category, low-risk patients receiving chemotherapy plus bevacizumab experienced a significantly improved median PFS of 13.5 months compared to 9.7 months with chemotherapy alone (*p* = 0.029; Figure 2a). High-risk patients also demonstrated a substantial PFS benefit with bevacizumab (13.9 months vs. 8.8 months, *p* < 0.001; Figure 2b). While bevacizumab did not significantly improve OS in low-risk patients (39.4 months vs. 33.3 months, *p* = 0.669; Figure 3a), a significant OS improvement was observed in high-risk patients (36.5 months vs. 23.2 months, *p* < 0.001; Figure 3b).

Multivariate Cox regression analysis identified risk group, recurrence site, and bevacizumab use as independent predictors of PFS. Specifically, high-risk patients exhibited a significantly elevated risk of disease progression compared to low-risk patients (hazard ratio [HR] 1.69; *p* = 0.007). Similarly, patients with distant recurrence had a significantly increased risk of progression compared to those with locoregional recurrence (HR: 1.58; *p* = 0.038). Conversely, the addition of bevacizumab to second-line chemotherapy was associated with a significantly reduced risk of disease progression (HR: 0.52; *p* = 0.001). The analysis of OS revealed that risk group, recurrence site, and surgical status were independent prognostic factors. The HR for OS in high-risk patients was 1.94 (*p* = 0.013), indicating a significantly increased risk of death compared to low-risk patients. Patients experiencing lymph node recurrence demonstrated a significantly higher risk of death (HR: 2.12; *p* = 0.030) compared to those with locoregional recurrence. Furthermore, patients who did not undergo optimal cytoreductive surgery had a significantly elevated risk of death (HR: 1.97; *p* = 0.045) compared to those who did (Table 3).

The incidence of any adverse event was 92.9% in the bevacizumab group compared to 83.9% in the chemotherapy-alone group (*p* = 0.062). Grade III or higher adverse events occurred more frequently in the bevacizumab group (32.1%) than in the chemotherapy group (17.7%, *p* = 0.040). Treatment discontinuation due to adverse events was also higher in the bevacizumab group (24.1%) compared to the chemotherapy group (8.1%, *p* = 0.009). Specific adverse events that were significantly more common in the bevacizumab group included hypertension (30.4% vs. 14.5%, *p* = 0.020), proteinuria (19.6% vs. 3.2%, *p* = 0.003), and epistaxis (35.7% vs. 11.3%, *p* = 0.001). There were no significant differences between the two groups for other side effects such as bone marrow suppression, venous thromboembolic events, or reduced left ventricular ejection fraction (Table 4).

## 3. Discussion

Our study demonstrated that high-risk patients with PSROC had significantly worse outcomes in terms of both PFS and OS compared to low-risk patients. In this patient group, compared to distant metastasis and lymph node metastasis, locoregional recurrence of the tumor and the addition of bevacizumab to chemotherapy were found to improve PFS, while the ability to perform optimal cytoreductive surgery, locoregional recurrence of the tumor, and a PFI of more than 12 months improved OS. Additionally, independent prognostic factors affecting PFS included risk group, recurrence location, and the addition of bevacizumab to chemotherapy. In contrast, independent prognostic factors influencing OS were identified as risk group, recurrence location, and surgical status.

Randomized controlled trials have provided significant data on the use of bevacizumab in women with PSROC [2,10,15]. The OCEANS study demonstrated that the addition of bevacizumab to carboplatin–gemcitabine chemotherapy significantly improved PFS in this patient group (12.4 months vs. 8.4 months; HR 0.48; *p* < 0.001) [15]. Similarly, the GOG 0213 study showed that combining bevacizumab with carboplatin–paclitaxel significantly increased PFS compared to chemotherapy alone (14.1 months vs. 10.3 months; HR 0.71; *p*: 0.0002). Although a numerical difference in OS was observed, it was not statistically significant (42.2 months vs. 39.3 months; HR 0.89, *p*: 0.19) [2]. The MITO-16B study, which included patients previously treated with bevacizumab, demonstrated that the addition of bevacizumab to carboplatin-based chemotherapy significantly improved PFS compared to carboplatin-based chemotherapy alone (13.8 months vs. 9.2 months; HR: 0.64; *p* < 0.0001). While adding bevacizumab was suggested to provide a potential survival advantage, this result was not statistically significant (30.9 months vs. 25.2 months; HR 0.80; *p* = 0.06) [16]. Meta-analyses evaluating the efficacy of the bevacizumab and chemotherapy combination in recurrent EOC have significantly improved PFS in platinum-sensitive patients [17]. A broader meta-analysis including phase 2 and 3 studies demonstrated that the bevacizumab combination therapy improved PFS (HR 0.73, *p* < 0.001) and reduced the risk of death (HR 0.83, *p* = 0.016) [18]. Another study examining the effects of bevacizumab over different follow-up periods observed improvements in PFS both in the short and long term (HR 0.71, *p* < 0.001 and HR 0.75, *p* < 0.001) but no significant difference in OS (HR 0.91, *p* = 0.06) [19]. Retrospective studies have yielded conflicting results regarding the impact of chemotherapy and bevacizumab on survival in PSROC. Some studies have shown that chemotherapy and bevacizumab improve PFS and OS [5,20], while others report no significant effect [21]. The results obtained in our study are similar to those of the OCEANS and GOG 0213 studies. Compared to chemotherapy alone, the chemotherapy plus bevacizumab arm showed a significant difference in PFS, but there was no difference in OS between the two groups.

These data suggest that while bevacizumab benefits PSROC, particularly regarding PFS, its effect on OS may vary depending on patient subgroups. In the ICON 7 and GOG 0218 studies, which evaluated the addition of bevacizumab to first-line chemotherapy in EOC, patients were stratified into risk groups based on prognostic factors in subgroup analyses. In the ICON 7 study, high-risk patients were stage IV, inoperable stage III, or stage III patients who underwent suboptimal debulking. In contrast, the GOG 0218 study defined high-risk patients as those with stage III disease who underwent suboptimal cytoreduction or stage IV disease [11,12].

In the ICON 7 study, the median OS was 30.2 months in the standard chemotherapy group compared to 39.7 months in the bevacizumab group (*p* = 0.03) among subgroups with a high risk of recurrence. However, no significant difference in OS was observed between the two treatment groups in the entire study population (45 months vs. 46 months, *p* = 0.85). Similarly, in low-risk patients, the median OS was 49.7 months in the standard chemotherapy group and 48.4 months in the bevacizumab group (HR: 1.14; *p* = 0.20). On the other hand, in high-risk patients, the median PFS was 10.1 months in the standard chemotherapy group and 15.6 months in the bevacizumab group (*p* = 0.005) [11].

In the GOG-218 study, no benefit in terms of OS was observed in the overall population (41 months vs. 43 months; HR 0.96). However, in the subgroup analysis by stage, an OS benefit was observed for stage IV patients (33 months vs. 43 months; HR 0.75) [22]. In a post hoc analysis of the GOG-0218 study, when high-risk patients were evaluated using the ICON7 criteria, an improvement in PFS was detected (median, 17.9 months vs. 10.7 months; HR: 0.63; *p* < 0.0001). However, no significant benefit in OS was found (median, 42.1 months vs. 38.6 months; HR: 0.86; *p* = 0.055). However, it should be noted that there are differences between these two studies, including the prognostic characteristics of the patient populations, bevacizumab doses, administration schedules, and post-progression treatment strategies [9]. These differences may contribute to explaining the discrepancies in the study results.

In our study, no difference in OS was observed between the treatment groups in the entire patient population (32.43 months vs. 36.53 months, *p* = 0.178). However, when patients were stratified into low-risk and high-risk groups, significant differences in survival were observed between the risk groups. Patients in the low-risk group exhibited significantly longer PFS and OS than those in the high-risk group. The median PFS was 13.7 months in the low-risk group and 10.8 months in the high-risk group (*p* = 0.007; Figure 1a). Similarly, the median OS was 36.5 months in the low-risk group and 23.5 months in the high-risk group (*p* = 0.003; Figure 1b). These findings support the prognostic value of risk stratification.

Among the low-risk patients, adding bevacizumab to chemotherapy significantly increased median PFS (13.5 months vs. 9.7 months, *p* = 0.029; Figure 2a). However, this PFS advantage did not translate into an OS benefit, with no significant difference observed in OS (39.4 months vs. 33.3 months, *p* = 0.669; Figure 3a). This suggests that while bevacizumab may improve disease control in low-risk patients, its impact on long-term survival may be limited. Conversely, in patients in the high-risk group, adding bevacizumab provided a marked benefit in both PFS and OS. The median PFS in patients receiving bevacizumab was 13.9 months, while it was 8.8 months in the group receiving chemotherapy alone (*p* < 0.001; Figure 2b). The median OS was 36.5 months in the bevacizumab group and 23.2 months in the control group (*p* < 0.001; Figure 3b). These results indicate that bevacizumab can significantly improve the disease course in high-risk patients.

These results parallel the findings of the previously conducted ICON7 and GOG-0218 studies. In these studies, while bevacizumab did not show a survival advantage in the general population, it provided benefits in specific subgroups, emphasizing the importance of accurately classifying the patient population when making treatment decisions. In our study, although no survival difference was observed between treatment groups in the overall patient population, adding bevacizumab significantly improved survival in high-risk patients. This finding aligns with the survival advantage observed in the high-risk patient group in the ICON7 study. Due to the more aggressive nature of their disease biology, patients may derive more significant benefits from anti-angiogenic therapy. Moreover, the lack of a survival advantage with the addition of bevacizumab in low-risk patients further underscores the need to consider the patient’s risk profile when making treatment decisions.

Recently, in EOC treatment studies, dividing the patient population into prognostic risk groups has become an increasingly common and contemporary approach in both clinical and research practice. Specifically, the identification of high-risk and low-risk patient subgroups allows for a more accurate assessment of treatment responses [14]. This approach is applied not only in studies evaluating anti-angiogenic agents but also in randomized controlled trials involving PARP inhibitors.

In the SOLO-1 trial conducted in patients with advanced EOC, patients who responded to platinum-based chemotherapy were compared with olaparib and placebo [23]. In the PAOLA-1 trial, the combination of olaparib and bevacizumab was compared only with bevacizumab alone [24]. In both studies, stage III patients who underwent interval cytoreductive surgery after neoadjuvant chemotherapy were classified into the high-risk group. In the PRIMA trial, patient selection was based on stricter criteria, as only patients who had received platinum-based neoadjuvant chemotherapy and exhibited stage IV disease during first-line treatment or had a significant residual tumor burden after primary cytoreduction were included.

According to the findings of the SOLO-1 trial, 42% of patients in the high-risk subgroup who received olaparib remained progression-free for 5 years compared to 17% in the placebo group. These rates were reported as 56% and 25% in the low-risk subgroup. In both groups, the median OS has not yet been reached [23]. In the PAOLA-1 trial, the combination of olaparib and bevacizumab demonstrated a significant benefit in PFS compared to bevacizumab alone in both the low-risk (HR: 0.46) and high-risk (HR: 0.60) subgroups [25].

However, the PRIMA and VELIA trials do not fully align with these findings [26,27]. In the PRIMA trial, patients with newly diagnosed advanced EOC who responded to first-line platinum-based chemotherapy and had multiple adverse prognostic factors were evaluated. In the overall study population, no significant difference in OS was observed between patients receiving niraparib and those receiving a placebo; the HR for OS was reported as 1.01, and the median OS durations were 46.6 and 48.8 months, respectively. Given the data, uncertainties remain regarding the optimal indications for combining PARP inhibitors and bevacizumab in the high-risk patient subgroup [27]. However, the results of phase 3 clinical trials and our current study support the notion that adding bevacizumab to platinum-based chemotherapy may be a practical approach in this group [11].

Bevacizumab inhibits angiogenesis by binding to isoforms of vascular endothelial growth factor A (VEGF-A) [28]. However, lymphangiogenesis also plays a role in tumor spread, and this process is regulated by factors such as VEGF-C and VEGF-D. These factors may function independently of bevacizumab’s mechanism of action. Therefore, the efficacy of bevacizumab remains uncertain, particularly regarding the position of residual or recurrent disease in lymph nodes and lymphatic vessels. Studies in PSROC have not precisely defined the localization of tumor recurrences, making it difficult to draw definitive conclusions about how this localization affects the efficacy of bevacizumab treatment [9].

Studies examining the relationship between lymph node metastasis in primary EOC and the outcomes of bevacizumab treatment have insufficient evidence. Similarly, current information on how bevacizumab’s efficacy in second-line treatment varies according to the localization of recurrence is limited [9].

Our study classified patients based on recurrence localization into locoregional, distant metastasis, and isolated lymph node involvement. In patients with locoregional recurrence, the median PFS was significantly higher than in those with distant metastasis or isolated lymph node involvement (*p* = 0.030). Conversely, patients with isolated lymph node involvement had substantially lower median OS than those with locoregional or distant recurrences (*p* = 0.011). These findings support the hypothesis that the efficacy of bevacizumab may be reduced in the presence of metastatic lymph node involvement in primary EOC [29]. However, further large-scale and confirmatory studies are needed to validate these findings and guide clinical practice.

Our study’s findings highlight the potential role of bevacizumab in treating platinum-sensitive recurrent ovarian cancer. The benefits observed with the addition of bevacizumab, particularly in high-risk patients, support the importance of personalized treatment approaches. In this context, a key question is how our findings can be translated into clinical practice. Current treatment guidelines already incorporate the use of bevacizumab in the management of recurrent ovarian cancer [30]. However, the differentiation of treatment decisions based on risk groups is not yet a widespread practice. Our findings may support the consideration of bevacizumab-containing regimens as a more aggressive treatment strategy in high-risk patients. Furthermore, considering the impact of recurrence location on treatment response, future clinical studies must optimize patient selection by accounting for this factor. As a concrete recommendation for clinicians, we suggest carefully evaluating patients’ risk factors and recurrence location, with treatment decisions shaped accordingly.

Optimal tumor resection is strongly associated with improved survival after cytoreductive surgery [3,15,16,31]. Numerous studies have demonstrated the positive impact of cytoreduction on survival. A meta-analysis revealed that patients undergoing optimal cytoreduction (with ≤1 cm residual disease) experienced better survival compared to those with suboptimal cytoreduction (>1 cm residual disease) (HR 1.36) [32]. Another meta-analysis found that patients with a maximal cytoreduction rate of less than 25% had a median survival of 22.7 months. In contrast, those with a 75% or higher rate had a median survival of 34 months [33]. Similarly, in our study, patients with optimal cytoreduction had a median OS of 36.53 months, those with suboptimal CRS had 31.58 months, and those without surgery had 18.83 months (*p* = 0.013). Another study assessing residual disease status in patients undergoing interval debulking surgery for advanced-stage EOC reported median PFS durations of 14 months for complete resection, 12 months for residual disease in one anatomical region, 10 months for multiple areas, and 6 months for suboptimal debulking. The median OS for these groups was 58 months, 37 months, 26 months, and 33 months, respectively [34]. These results support the significant impact of the amount of residual disease obtained in cytoreductive surgery on patient prognosis.

In our study, when comparing chemotherapy and chemotherapy + bevacizumab groups, bevacizumab-related adverse effects (hypertension, proteinuria, and epistaxis) were significantly more frequent in the chemotherapy + bevacizumab group. Additionally, grade III or higher adverse events and those leading to treatment discontinuation were significantly higher in the bevacizumab group.

These findings align with other clinical studies evaluating the use of bevacizumab in PSROC [2,10]. These studies reported an increase in the incidence of hypertension and proteinuria in bevacizumab-treated groups, but the treatment was generally considered tolerable. Similarly, in our study, despite the increased frequency of adverse events, the continuation rates of treatment were high, suggesting that bevacizumab can be used safely with proper patient selection.

However, our study has certain limitations. Although the retrospective design has the potential for selection bias, it provides valuable insights by reflecting real-world data. Being a single-center study limits its generalizability, but it allowed for detailed analysis within a homogeneous patient group. The data obtained from hospital records may contain some missing or inaccurate information, which could limit the analysis; however, significant clinical conclusions have been drawn based on the large dataset. Variations in treatment regimens based on patient and physician preferences and the absence of a standardized protocol for bevacizumab duration could influence the results. However, this variability better reflects actual clinical practice. Additionally, although next-generation PARP inhibitors and other targeted agents were excluded from the evaluation, the findings of this study may provide insight for future research investigating the efficacy of combined therapies.

## 4. Materials and Methods

This retrospective study includes patients diagnosed with EOC who presented to our center between January 2016 and December 2023. The inclusion criteria were International Federation of Gynecology and Obstetrics (FIGO) stage III and IV disease, adequate liver, kidney, and cardiac function, and PSROC. Patients who received bevacizumab during primary treatment were excluded. Exclusion criteria included a history of malignancy other than EOC, prior bowel perforation or gastrointestinal complications, and an Eastern Cooperative Oncology Group performance score of 3 or 4. The study was conducted in accordance with the principles of ethics. It was approved by the T.C. Ministry of Health’s Dr. Abdurrahman Yurtaslan, Ankara Oncology Training and Research Hospital Non-Interventional Clinical Research Ethics Committee (No: 2025-01/06). The ethics committee waived the requirement for informed consent.

Demographic and clinical data were retrospectively obtained from patients’ electronic medical records and charts. Included were Eastern Cooperative Oncology Group (ECOG) performance status, germline *BRCA* mutation status, tumor stage, histological subtype, the presence of ascites at recurrence, primary cytoreductive surgery status, primary tumor location, recurrence location, chemotherapeutic agents administered concurrently with carboplatin at recurrence, platinum-free interval (PFI), CA-125 levels at diagnosis, and CA-125 levels at recurrence. Germline *BRCA* mutation analysis was performed using next-generation sequencing (NGS). Samples with negative NGS results underwent further analysis using multiplex ligation-dependent probe amplification.

Platinum-sensitive recurrence is defined as disease recurrence occurring after a remission period of at least six months following the completion of the last platinum-based chemotherapy regimen [10]. Patients were divided into three groups according to the location of recurrence: isolated lymph node metastasis, locoregional recurrence, and distant metastatic disease. Patients were divided into two groups according to PFI: 6–12 months and >12 months; for patients who underwent primary cytoreductive surgery (CRS), residual disease status is classified as optimal (maximum tumor diameter ≤1 cm) or suboptimal (maximum tumor diameter >1 cm) as defined by the Gynecologic Oncology Group [31,35]. Patients were grouped according to median CA-125 levels at diagnosis and at recurrence.

Patients were divided into two risk groups to compare their baseline characteristics: high risk and low risk. Patients with FIGO stage 4 and those with FIGO stage 3 who underwent suboptimal CRS or were inoperable were considered high risk. In contrast, patients with stage 3 who underwent optimal CRS were considered low-risk [11].

Chemotherapy regimens include paclitaxel, gemcitabine, or liposomal doxorubicin in combination with carboplatin. Carboplatin was administered at 4–6 AUC based on the creatinine clearance rate, paclitaxel at 175 mg/m^2^, gemcitabine at 1000 mg/m^2^ (days 1 and 8), and liposomal doxorubicin at 40–50 mg/m^2^ every three weeks. Patients in both groups received bevacizumab intravenously at a dose of 15 mg/kg every three weeks [2,10,16]. In patients receiving bevacizumab, treatment was discontinued after 4–6 cycles of chemotherapy, and bevacizumab maintenance therapy was continued in the absence of disease progression, unacceptable toxicity, physician preference, or patient request, unless there were bevacizumab-related complications. Patients receiving chemotherapy alone discontinued treatment after 4–6 cycles.

Descriptive statistics for categorical variables were presented as frequencies and percentages. Patients were divided into two groups: high risk and low risk. Continuous variables were compared between the two groups using the independent samples t-test or the Mann–Whitney U test, as appropriate. Categorical variables were compared using the chi-square test or Fisher’s exact test. PFS was the time from treatment initiation to disease progression, last follow-up, or death from any cause. OS was defined as the time from diagnosis to death from any cause or the last follow-up. Both PFS and OS were calculated using the Kaplan–Meier method. The two groups used the Kaplan–Meier method and log-rank test to compare PFS and OS. A Cox proportional hazards regression model was constructed using factors identified as statistically significant in the Kaplan–Meier analysis to determine independent prognostic factors. All tests were two-sided; a *p*-value of less than 0.05 was considered statistically significant. Statistical analyses were performed using IBM SPSS Statistics for Windows, version 25.0.

## 5. Conclusions

Our study demonstrates that the addition of bevacizumab provides significant benefits, particularly in high-risk patients with platinum-sensitive recurrent ovarian cancer. While risk-group-based subgroup analyses exist in the literature for newly diagnosed epithelial ovarian cancer patients, our study is the first to evaluate bevacizumab efficacy by performing clinical risk stratification in patients with PSROC. Although the findings need to be validated in larger patient cohorts and prospective studies, the combination of bevacizumab with chemotherapy should be considered a promising treatment strategy in high-risk EOC patients. The positive impact of optimal cytoreduction on survival and the variability of bevacizumab efficacy according to recurrence location highlight the need to evaluate patients’ clinical characteristics in more detail when developing treatment strategies. In conclusion, this study makes a unique and valuable contribution to the literature by assessing the role of bevacizumab in a risk-based and recurrence-specific context. The findings support the development of more personalized treatment approaches for PSROC in the future.

## Figures and Tables

**Table 1:** Characteristics of the patients at baseline.

Factor	Total n = 174 (%)	Low Risk n = 102 (58.6%)	High Risk n = 72 (41.4%)	*p*-Value
Age-year *–median				0.130
≤58	94 (54.0%)	60 (58.8%)	34 (47.2%)	
>58	80 (46.0%)	42 (41.2%)	38 (52.8%)	
ECOG performance status *				<0.001
0–1	148 (85.1%)	97 (95.1%)	51 (70.8%)	
2	26 (14.9%)	5 (4.9%)	21 (29.2%)	
*gBRCAm*				0.946
1 or 2 mutants	32 (18.4%)	18 (17.6%)	14 (19.4%)	
Wild	45 (25.9%)	27 (26.5%)	18 (25.0%)	
Unknown	97 (55.7%)	57 (55.9%)	40 (55.6%)	
FIGO Stage *				<0.001
III	134 (77.0%)	102 (100.0%)	32 (45.2%)	
IV	40 (23.0%)	0 (0.0%)	40 (54.8%)	
Histology *				0.136
High-grade serous	130 (74.7%)	72 (70.6%)	58 (80.6%)	
Other or unknown	44 (25.3%)	30 (29.4%)	14 (19.4%)	
Ascites				0.681
Yes	119 (68.4%)	71 (69.6%)	48 (66.7%)	
No	55 (31.6%)	31 (30.4%)	24 (33.3%)	
Cytoreductive surgery *				<0.001
Suboptimal	43 (24.7%)	0 (0.0%)	43 (59.7%)	
Optimal	108 (62.1%)	102 (100.0%)	6 (8.4%)	
Non-operable	23 (13.2%)	0 (0.0%)	23 (31.9%)	
Location of recurrence				0.916
Local	108 (62.1%)	62 (60.8%)	46 (63.9%)	
Distant	48 (27.6%)	29 (28.4%)	19 (26.4%)	
Lymph node	18 (10.3%)	11 (10.8%)	7 (9.7%)	
Second-line agents				0.451
Chemotherapy	62 (35.6%)	34 (33.3%)	28 (38.9%)	
Chemotherapy Plus bevacizumab	112 (64.4%)	68 (66.7%)	44 (61.1%)	
Maintenance bevacizumab therapy				
Yes	48 (42.9%)	33 (48.5%)	15 (34.1%)	0.132
No	64 (57.1%)	35 (51.5%)	29 (65.9)	
Platinum-free interval				0.199
6–12 months	89 (51.1%)	48 (47.1%)	41 (56.9%)	
>12 months	85 (48.9%)	54 (52.9%)	31 (43.1%)	
Median CA-125 levels at diagnosis				0.163
≤500	81 (46.6%)	52 (51.0%)	29 (40.3%)	
>500	93 (53.4%)	50 (49.0%)	43 (59.7%)	
Median CA-125 levels at recurrence				0.742
≤100	92 (52.9%)	55 (53.9%)	37 (51.4%)	
>100	82 (47.1%)	47 (46.1%)	35 (48.6%)	

ECOG: Eastern Cooperative Oncology Group; *gBRCAm*: germline *BRCA1/BRCA2* mutation; FIGO: International Federation of Gynecology and Obstetrics; * before starting treatment for platinum-sensitive recurrence.

**Table 2:** PFS and OS times in patients with PSROC.

Factor	PFS Median (95% CI)	*p*-Value	OS Median (95% CI)	*p*-Value
Age median (range) ^1^		0.133		0.433
≤58	13.21 (11.46–14.96)		34.73 (20.22–39.24)	
>58	11.20 (9.98–12.43)		30.65 (26.67–34.63)	
FIGO Stage ^1^		0.386		0.986
III	12.03 (10.54–13.51)		34.14 (29.63–38.65)	
IV	11.54 (8.88–14.20)		31.58 (26.27–36.89)	
Histology ^1^		0.147		0.776
High-grade serous	12.55 (11.40–13.70)		32.81 (27.87–37.76)	
Other or unknown	9.68 (7.83–11.53)		32.43 (29.21–35.65)	
Ascites		0.090		0.912
No	11.54 (9.83–13.25)		32.43 (28.79–36.06)	
Yes	12.03 (5.42–18.63)		34.73 (31.71–37.75)	
Risk groups		0.007 *		0.003 *
Low risk	13.73 (12.94–14.52)		36.53 (32.59–40.46)	
High risk	10.81 (8.75–12.87)		23.49 (18.41–28.58)	
Cytoreductive surgery ^1^		0.056		0.013 *
Suboptimal	11.34 (10.72–11.95)		31.58 (24.43–38.72)	
Optimal	13.21 (12.31–14.10)		36.53 (32.61–40.44)	
Non-operable	6.97 (5.43–8.51)		18.83 (11.14–26.51)	
Location of recurrence		0.030 *		0.011 *
Locoregional	13.04 (12.16–13.93)		34.73 (31.54–37.92)	
Distant	10.58 (9.34–11.82)		31.58 (25.00–38.16)	
Lymph node	10.76 (6.68–14.84)		21.20 (12.07–30.33)	
Second-line treatment		<0.001 *		0.178
Chemotherapy	9.68 (8.31–11.05)		32.43 (27.98–36.88)	
Chemotherapy Plus bevacizumab	13.73 (12.30–15.17)		36.53 (30.41–42.64)	
Maintenance bevacizumab therapy		0.114		0.914
Yes	15.93 (12.97–18.90)		36.53 (26.83–46.23)	
No	12.03 (10.05–14.00)		31.58 (16.80–46.36)	
Platinum-free interval		0.226		0.014 *
6–12 months	10.81 (9.67–11.95)		27.92 (20.91–34.92)	
>12 months	12.81 (11.85–13.77)		36.53 (30.22–42.83)	
Median CA-125 levels at diagnosis		0.769		0.867
≤500	12.77 (11.37–14.17)		32.43 (28.00–36.85)	
>500	11.54 (10.29–12.79)		34.14 (29.88–38.40)	
Median CA-125 levels at recurrence		0.561		0.119
≤100	12.55 (9.76–15.34)		39.26 (28.75–49.97)	
>100	11.34 (10.11–12.56)		32.43 (27.76–37.09)	

PSROC: platinum-sensitive recurrent ovarian cancer; PFS: progression-free survival; OS: overall survival; CI: confidence interval; FIGO: International Federation of Gynecology and Obstetrics; ^1^ before starting treatment for platinum-sensitive recurrence; * significant.

**Table 3:** Cox regression model for predicting the independent factors for PFS and OS.

	PFS		OS	
	HR (95%CI)	*p*-Value	HR (95%CI)	*p*-Value
Risk groups				
Low Risk	Ref		Ref	
High Risk	1.69 (1.16–2.46)	0.007 *	1.94 (1.15–3.26)	0.013 *
Location of recurrence				
Locoregional	Ref		Ref	
Distant	1.58 (1.03–2.42)	0.038 *	1.50 (0.93–2.42)	0.099
Lymph node	1.60 (0.86–2.98)	0.138	2.12 (1.07–4.17)	0.030 *
Second-line treatment				
Chemotherapy	Ref		-	
Chemotherapy Plus bevacizumab	0.52 (0.36–0.77)	0.001 *	-	
Platinum-free interval		-		
6–12 months	-		Ref	
>12 months	-		0.69 (0.46–1.04)	0.076
Cytoreductive surgery ^1^		-		
Suboptimal	-		Ref	
Optimal	-		1.20 (0.67–2.13)	0.544
Non-operable	-		1.97 (1.02–3.83)	0.045 *

PFS: progression-free survival; OS: overall survival; HR: hazard ratio; CI: confidence interval; ^1^ before starting treatment for platinum-sensitive recurrence; * significant.

**Table 4:** Adverse events in PSROC treated with chemotherapy alone versus chemotherapy plus bevacizumab.

	Chemotherapy Plus Bevacizumab (n = 112)	Chemotherapy (n = 62)	*p*-Value
Any adverse events	104 (92.9%)	52 (83.9%)	0.062
Adverse event of grade III or higher	36 (32.1%)	11 (17.7%)	0.040 *
Adverse events that led to treatment discontinuation	27 (24.1%)	5 (8.1%)	0.009 *
Neutropenia	87 (77.7%)	46 (74.2%)	0.604
Thrombocytopenia	58 (51.8%)	35 (56.5%)	0.555
Anemia	77 (68.8%)	48 (77.4%)	0.223
Hypertension	34 (30.4%)	9 (14.5%)	0.020 *
Proteinuria	22 (19.6%)	2 (3.2%)	0.003 *
Venous thromboembolic event	12 (10.7%)	3 (4.8%)	0.186
Decreased left ventricular ejection fraction	9 (8.0%)	3 (4.8%)	0.425
Fistula	2 (1.8%)	0 (0%)	0.290
Increased serum creatinine	18 (16.1%)	6 (9.7%)	0.241
Epistaxis	40 (35.7%)	7 (11.3%)	0.001 *
Gastrointestinal perforation	1 (0.9%)	0 (0%)	0.456
PRES	0 (0%)	0 (0%)	NA

PRES: posterior reversible encephalopathy syndrome; * significant.

## Data Availability

The datasets used in this study are not available for sharing in accordance with institutional policies and to protect patient confidentiality.

## Abbreviations

The following abbreviations are used in this manuscript:

EOC	Epithelial ovarian cancer
PSROC	Platinum-sensitive recurrent ovarian cancer
VEGF	Vascular endothelial growth factor
PARP	Poly(ADP-ribose) polymerase
CRS	Cytoreductive surgery
HRD	Homologous recombination deficiency
FIGO	International Federation of Gynecology and Obstetrics
PFS	Progression-free survival
OS	Overall survival
PFI	Platinum-free interval
HR	Hazard ratio
CI	Confidence interval
